# Plasma from human volunteers subjected to remote ischemic preconditioning protects human endothelial cells from hypoxia–induced cell damage

**DOI:** 10.1007/s00395-015-0474-9

**Published:** 2015-02-26

**Authors:** Nina C. Weber, Isabelle Riedemann, Kirsten F. Smit, Karina Zitta, Djai van de Vondervoort, Coert J. Zuurbier, Markus W. Hollmann, Benedikt Preckel, Martin Albrecht

**Affiliations:** 1 Laboratory of Experimental Intensive Care and Anaesthesiology (L.E.I.C.A.), Department of Anaesthesiology, University of Amsterdam, Academic Medical Centre (AMC), Meibergdreef 9, 1100 DD Amsterdam, The Netherlands; 2 Department of Anaesthesiology and Intensive Care Medicine, University Hospital Schleswig-Holstein, Campus Kiel, Kiel, Germany

**Keywords:** Remote conditioning, Human endothelium, Signalling kinases, Translational study

## Abstract

Short repeated cycles of peripheral ischemia/reperfusion (I/R) can protect distant organs from subsequent prolonged I/R injury; a phenomenon known as remote ischemic preconditioning (RIPC). A RIPC-mediated release of humoral factors might play a key role in this protection and vascular endothelial cells are potential targets for these secreted factors. In the present study, RIPC-plasma obtained from healthy male volunteers was tested for its ability to protect human umbilical endothelial cells (HUVEC) from hypoxia–induced cell damage. 10 healthy male volunteers were subjected to a RIPC-protocol consisting of 4 × 5 min inflation/deflation of a blood pressure cuff located at the upper arm. Plasma was collected before (*T*0; control), directly after (*T*1) and 1 h after (*T*2) the RIPC procedure. HUVEC were subjected to 24 h hypoxia damage and simultaneously incubated with 5 % of the respective RIPC-plasma. Cell damage was evaluated by lactate dehydrogenase (LDH)-measurements. Western blot experiments of hypoxia inducible factor 1 alpha (HIF1alpha), phosphorylated signal transducer and activator of transcription 5 (STAT5), protein kinase B (AKT) and extracellular signal-related kinase 1/2 (ERK-1/2) were performed. Furthermore, the concentrations of hVEGF were evaluated in the RIPC-plasma by sandwich ELISA. Hypoxia–induced cell damage was significantly reduced by plasma *T*1 (*p* = 0.02 vs *T*0). The protective effect of plasma *T*1 was accompanied by an augmentation of the intracellular HIF1alpha (*p* = 0.01 vs *T*0) and increased phosphorylation of ERK-1/2 (*p* = 0.03 vs *T*0). Phosphorylation of AKT and STAT5 remained unchanged. Analysis of the protective RIPC-plasma *T*1 showed significantly reduced levels of hVEGF (*p* = 0.01 vs *T*0). RIPC plasma protects endothelial cells from hypoxia–induced cell damage and humoral mediators as well as intracellular HIF1alpha may be involved.

## Introduction

Transient episodes of ischemia (ischemic preconditioning), if applied before prolonged ischemia/reperfusion injury, are organ protective [12, 26]. Ischemic preconditioning does not only act locally, but is also able to protect remote tissues from ischemia/reperfusion injury, a phenomenon described as remote ischemic preconditioning (RIPC). RIPC can be induced by inflation and deflation of a blood pressure cuff located at the upper or lower limb. This procedure has been shown to attenuate organ injury in a number of experimental and clinical situations [26, 27, 58].

Despite the encouraging results of RIPC in preclinical and animal studies, a translation of RIPC into the clinic is still not fully achieved [2, 16, 49, 57]. However, there are several clinical trials in progress elucidating the potential clinical benefit of RIPC. In patients undergoing coronary artery bypass graft (CABG) surgery an acute and probably favourable attenuation of cardiac enzyme release has been demonstrated, and thus there is evidence that RIPC provides perioperative myocardial protection and improves the prognosis of these patients [9, 68, 69]. Similar protective effects could also be found in patients undergoing elective percutaneous coronary intervention (PCI) [14, 34] and ST-elevation myocardial infarction (STEMI) patients undergoing primary PCI [5, 65]. On the other hand, deleterious effects with a significant increase in cardiac injury have been described [10, 37, 59]. Therefore, the results of the large clinical trials ongoing in CABG patients [22, 50] will substantially increase our knowledge about the translatability of RIPC in the clinical setting.

The underlying mechanisms of RIPC have been attributed to humoral, neuronal and anti-inflammatory pathways [27]. However, the exact mechanisms are complex and yet not completely understood [24, 62]. It is suggested that the humoral mediators might be released from the remote tissue into the blood stream from where they are transported to the target organ, and/or that they are produced after stimulation via neuronal pathways in the target organ itself [24, 47]. In the last years, several so far unidentified mediators of RIPC have been described by different groups: e.g. stomal derived factor (SDF) 1alpha [6], exosomes [19], Apolipoprotein A1 [32], miR144 [45], IL-10 [8], matrix metalloproteinases (MMPs) [74] and nitrite [60]. For review, also see [27].

For several reasons the vascular endothelium, especially in the target organ, could play a central role in the RIPC-mediated mechanisms of organ protection from I/R injury: (1) humoral factors that are released into the blood stream upon RIPC stimulus may directly interact with endothelial cells which in turn may directly or indirectly transfer the RIPC stimulus to the underlying tissue [51]. (2) Endothelial cells are among the first cell types that will encounter hypoxia in the target organ and respond to it [63]. (3) Endothelial dysfunction is a major reason for severe local and systemic consequences of I/R injury [63]. Moreover, it has been demonstrated that RIPC before primary percutaneous coronary intervention significantly improves endothelial function in patients with acute myocardial infarction, and this effect remains constant for at least a week [48]. These data suggest that the improvement of endothelial function may be one possible explanation for the protective effects of RIPC. In this context it is worth mentioning that the coronary circulation and cardiac remodelling that are directly related to the endothelial function just recently have been recognized to be critical determinants of cardioprotective interventions [28, 29].

In the present study, RIPC-plasma was obtained from healthy male volunteers and tested for its ability to protect human vascular endothelial cells (HUVEC) from hypoxia–induced cell damage. We furthermore investigated the cellular target mechanisms that were affected by RIPC-plasma in HUVEC cells subjected to a hypoxic insult.

## Materials and methods

### Chemicals, solutions and culture media

If not otherwise stated all chemicals and solutions were purchased either from Roche (Almere, the Netherlands), Sigma-Aldrich (Zwijndrecht, Netherlands), Merck (Millipore, Amsterdam, Netherlands) or Carl Roth (Karlsruhe, Germany).

### Isolation of human umbilical vein endothelial cells (HUVEC)

HUVEC were freshly isolated from umbilical cords as described previously [71] (Waiver: W12-167#12.17.096, Ethical committee Amsterdam) and maintained in a humidified atmosphere of 5 % carbon dioxide/95 % air at 37 °C in ECGM medium supplemented with endothelial cell growth supplement (Promocell Bio-Connect, Huizen, Netherlands), 1 % penicillin/streptomycin, 1 % amphotericin B and 10 % heat-inactivated foetal bovine serum (PAA, Germany, Freiburg). All culture surfaces were coated with 0.75 % gelatine (BD Diagnostic Systems, Netherlands, Breda) prior to cell seeding. Only cells from passage 3 were used in the experiments. Characterization of HUVEC cells was performed by detection of von Willebrand-factor by fluorescence staining and fluorescence activated cell sorting FACS (data not shown).

### Collection of human plasma

The study was approved by the local ethics committee of the Academic Medical Centre (AMC), University of Amsterdam, The Netherlands, (ISRCTN59201440) and was performed in accordance with the Declaration of Helsinki and the Medical Research Involving Human Subjects Act. Ten healthy male volunteers (25.20 ± 3.39 years) were subjected to a RIPC-protocol consisting of 4 × 5 min inflation/deflation of a blood pressure cuff located at the upper right arm. Prior to participation, all subjects gave their written informed consent. The following inclusion criteria were chosen: (1) healthy, (2) male, (3) age between 18 AND 45 years. Exclusion criteria were as follows: (1) cardiovascular, kidney, pulmonary or endocrine diseases, (2) alcohol or drug abuse, and (3) no informed consent. We decided to only include male volunteers into the study to avoid potential influences of oestrogens [56]. Subject characteristics are given in Table 1.

**Table 1:** Summarized volunteer data

Volunteer identification #	1	2	3	4	5	6	7	8	9	10	Mean	SD
Age [years]	26	21	19	27	28	24	27	23	30	27	25.20	3.39
Height [m]	1.80	1.85	1.85	1.95	1.90	1.82	1.93	1.80	1.86	1.75	1.85	0.06
Weight [kg]	74	85	80	95	85	73	95	70	73	78	80.80	9.02

**Fig. 1:**
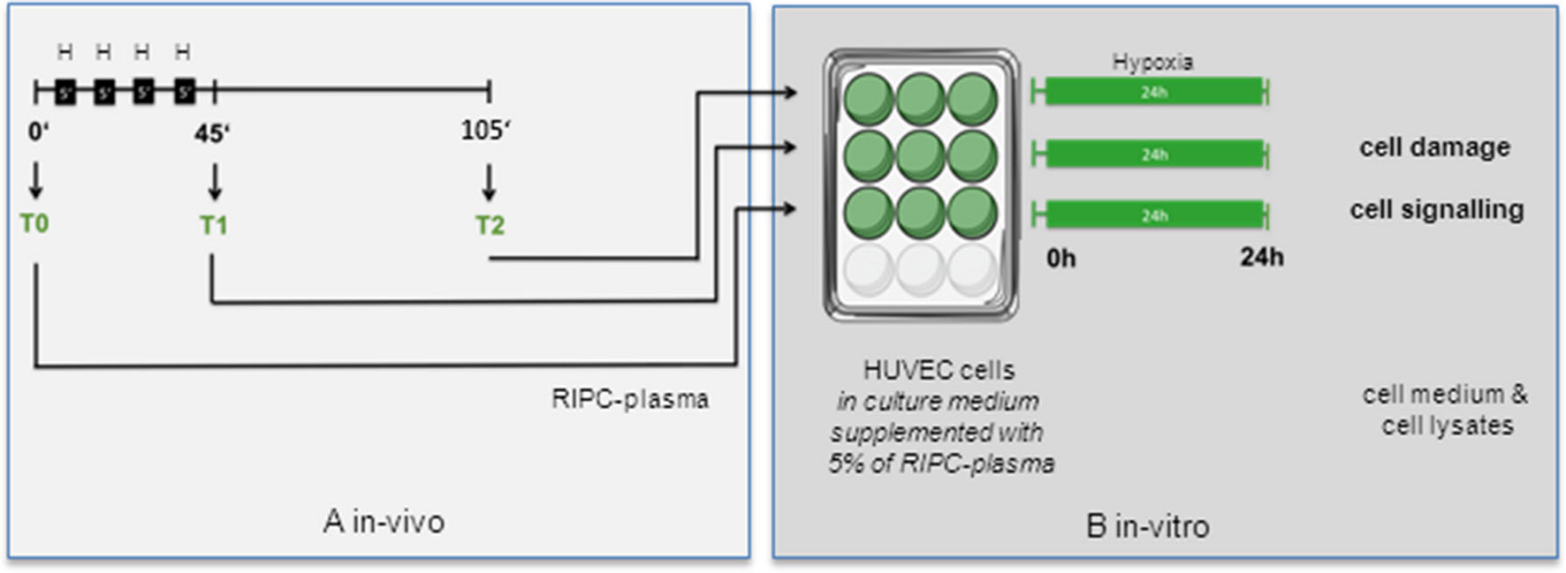
Experimental setting. The RIPC-protocol consisted of 4 × 5 min inflation/deflation of a blood pressure cuff. Plasma was obtained before (*T*0), directly after (*T*1) and 60 min after (*T*2) the RIPC stimulus. HUVEC cells were incubated with the respective plasma and subjected to 24 h of hypoxia. Employing cell culture media and cell lysates, cell damage as well as cellular signalling events were investigated. *H* hypoxia; *green color*, RIPC-plasma

Blood was collected before (T0; baseline time point prior to inflation of blood pressure cuff = controls), directly after (*T*1) and 60 min after (*T*2) the RIPC-protocol (Fig. 1). The blood samples were collected in citrate vials (BD bioscience, Breda, The Netherlands) and centrifuged at 4 °C, 290 g for 10 min. Plasma was aliquoted, and stored at −80 °C.

To evaluate the optimal concentration of human plasma to be added to the culture medium, HUVEC cells were incubated with different concentrations (0, 1, 5, 10 %) of fetal bovine serum and growth characteristics as well as the degree of hypoxia–induced cell damage were evaluated. On the basis of these results, the optimal concentration of plasma was selected. A similar approach has also been chosen in our recently published study in which RIPC serum was used [74]. These evaluations suggested that the basic culture medium should be supplemented with 5 % plasma.

### Induction of in vitro hypoxia in HUVEC by enzymatic oxygen depletion

Hypoxic conditions in HUVEC were induced using a modified version of our recently described enzymatic model [36, 75]. In the present study concentrations of the hypoxia inducing enzymes were adapted to 4 U/ml glucose oxidase (GO), and 120 U/ml catalase (CAT) and cell culture plates were transferred into an airtight chamber, which was flooded with 1 bar, 10 l/min nitrogen gas until oxygen was reduced to 1 %. The box was then kept inside an incubator at 37 °C. Oxygen levels were monitored directly in the culture medium using an OxyMini fibre optic oxygen meter (World Precision Instruments, USA, Sarasota) and OxyMicro Software v.00 04/2003.

### Experimental protocol

HUVEC cells were seeded into gelatine-coated 12 well plates 2 days before the experiment. At the beginning of the experiment ECGM growth medium was exchanged and cells were pre-incubated for 1 h with M199 medium (PAN Biotech, Aidenbach, Germany) containing 5 % of the respective RIPC-plasma (*T*0, *T*1, *T*2). Subsequently, the normoxic medium was replaced by hypoxic M199 containing 5 % of the respective RIPC-plasma and the culture plates were placed in an airtight chamber and kept inside an incubator for 24 h. For colorimetric lactate dehydrogenase (LDH)-measurements the cell culture medium of each well was collected after 24 h of hypoxia and frozen at −20 °C. For Western blotting experiments the cells were harvested and frozen at −80 °C (Fig. 1).

### Determination of cell death

LDH activity was colorimetrically evaluated using a Lactate Dehydrogenase Activity Assay Kit (Biovision, Uithoorn, Netherlands,) following the manufacturer’s protocol. Absorbance was measured before and after 30 min of incubation (37 °C) at 450 nm using an ELISA reader (Tecan, Crailsheim, Germany).

### Western blotting

Western blotting was performed as described previously [74]. Overnight incubation of the membranes was conducted at 4 °C with appropriate dilutions of primary antibodies directed against HIF1alpha (Acris, Novus Biological, Herford, Germany, 1:1000), actin (Santa Cruz, Heidelberg, Germany, 1:1000), pAKT (Cell Signalling, Danvers, USA, 1:1000), AKT (Cell Signalling, Danvers, USA, 1:2000), pERK-1/2 (Cell Signalling, Danvers, USA, 1:8000), ERK-1/2 (Cell Signalling, Danvers, USA, 1:8000), pSTAT5 (R&D Systems, Wiesbaden-Nordenstadt, Germany, 1:1000), or against STAT5 (R&D Systems, Wiesbaden-Nordenstadt, Germany, 1:1000). Membranes were rinsed three times for 10 min with tris-buffered saline plus tween buffer (TBST) at room temperature, and were—depending on the primary antibody used—incubated for 1 h with the secondary horseradish peroxidase coupled-antibody (anti-rabbit, DAKO, Eching, Germany, 1:10,000 or anti-goat, Santa Cruz, Heidelberg, Germany, 1:10,000), with a biotin coupled-antibody (anti-rabbit, Abcam, Cambridge, UK, 1:10,000), or with horseradish peroxidase-coupled streptavidin for the biotinylated secondary antibody (AbD Serotec, Puchheim, Germany, 1:5000). After washing three times with TBST buffer for 10 min, the membranes were incubated with ECL detection reagent (GE Healthcare Life Sciences, Freiburg, Germany) for 5 min. For signal detection, autoradiography films (GE Healthcare Life Sciences, Freiburg, Germany) were exposed to the membranes for various time periods in the dark. Relative intensities of protein bands were analysed by ImageJ v1.48 and GraphPad Prism 5.0 for Mac. In some experiments, membranes were stripped and re-probed with different antibodies. Therefore, the membranes were incubated for 15 min at 56 °C with stripping buffer (4 ml SDS 10 %, 2.5 ml Tris 0.5 M pH 6.8, 13.5 ml ultrapure water, 160 µl 2-mercaptoethanol). After washing with TBST buffer, the membranes were re-blocked with 3 % BSA/TBST buffer for 1 h and were washed three times with TBST buffer.

### Quantification of human vascular endothelial growth factor

The concentrations of the human Vascular Endothelial Growth Factor (hVEGF) were determined in RIPC-plasma by specific ELISA systems, (ScienceCell Research Laboratories, USA, Carlsbad) according to the manufacturer’s protocol. Absorbance was measured at 450 nm using an ELISA reader (Tecan, Germany, Crailsheim) and hVEGF concentrations were calculated from the standard curve provided.

### Statistical analysis

Statistics were performed using the software GraphPad Prism 5.0 for Mac. D’Agostino normality testing was used to check data for normal distribution. Parametric data were analysed using One-Sample *t*-Tests (LDH-activity, hVEGF) or Paired *t*-Tests (HIF1alpha, pSTAT5). Non-parametric data were analysed using the Wilcoxon signed rank test (pAKT, pERK-1/2). Variables are expressed as mean ± SEM.

## Results

### Plasma obtained directly after RIPC reduces the hypoxia–induced cell damage in HUVEC cells

**Fig. 2:**
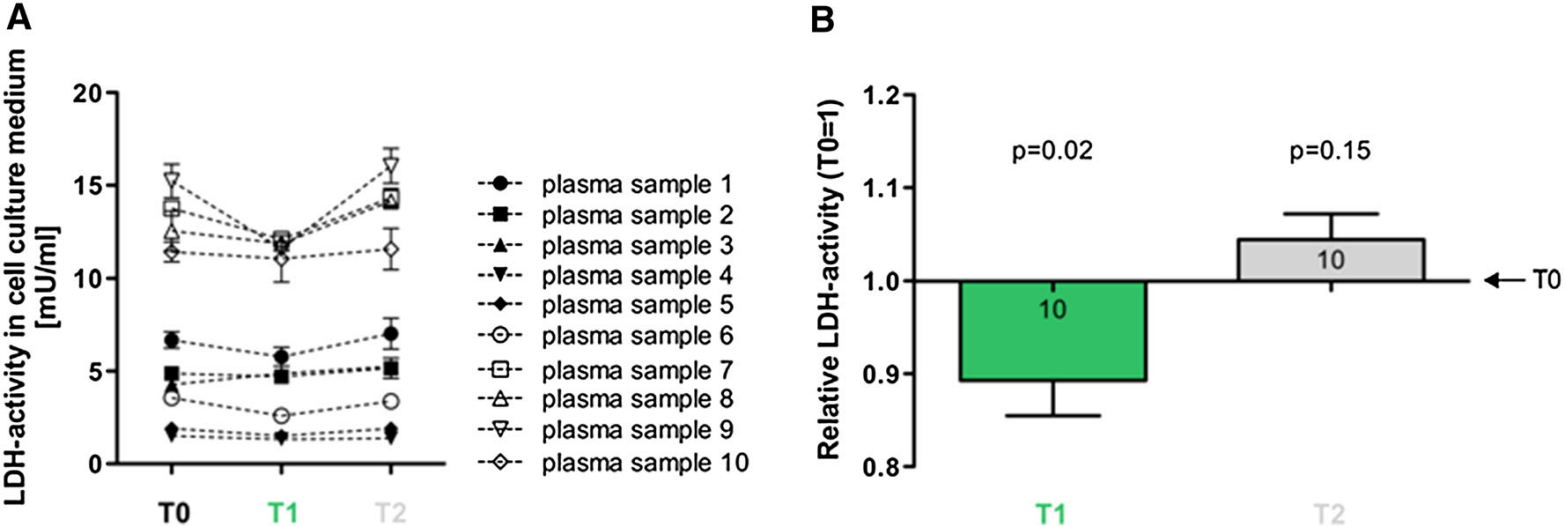
Effects of RIPC-plasma on hypoxia–induced damage of HUVEC cells. **a** Quantification of LDH-activities as a marker for cell damage in HUVEC cell culture media. Culture media were supplemented with 5 % plasma (*T*0, *T*1 and *T*2) from the various volunteers (#1–#10) and LDH-activities were measured after 24 h of hypoxia. **b** Relative LDH-activities in culture media of HUVEC cells after 24 h of hypoxia. Cell culture media were supplemented with plasma *T*0, *T*1 or *T*2. Hypoxia–induced cell damage is significantly reduced by the addition of plasma *T*1. *Numbers* *in the columns* show the numbers of different plasma samples used. *Columns* display the mean ± SEM

LDH-assays were used to evaluate the influence of RIPC-plasma (*T*1, *T*2) on hypoxia–induced cell damage of HUVEC cells. *T*0-plasma, obtained prior to RIPC, was used as baseline control. Mean LDH-activity in the culture media at baseline was 7.58 ± 0.9 mU/ml, while the range of LDH activity varied between different samples (minimum LDH: 1.50 mU/ml, maximum LDH: 15.24 mU/ml; Fig. 2a) Compared to plasma *T*0, plasma *T*1 significantly reduced the hypoxia–induced cell damage (*T*1: 0.89 ± 0.04; *T*0 = 1; *p* = 0.02; Fig. 2b). Plasma *T*2 did not significantly change the hypoxia induced damage in HUVEC cells (*T*2: 1.04 ± 0.03; *T*0 = 1; *p* = 0.15; Fig. 2b).

### The reduction of hypoxia–induced cell damage by RIPC-plasma is associated with an increased protein expression of HIF1alpha and enhanced phosphorylation of ERK-1/2

**Fig. 3:**
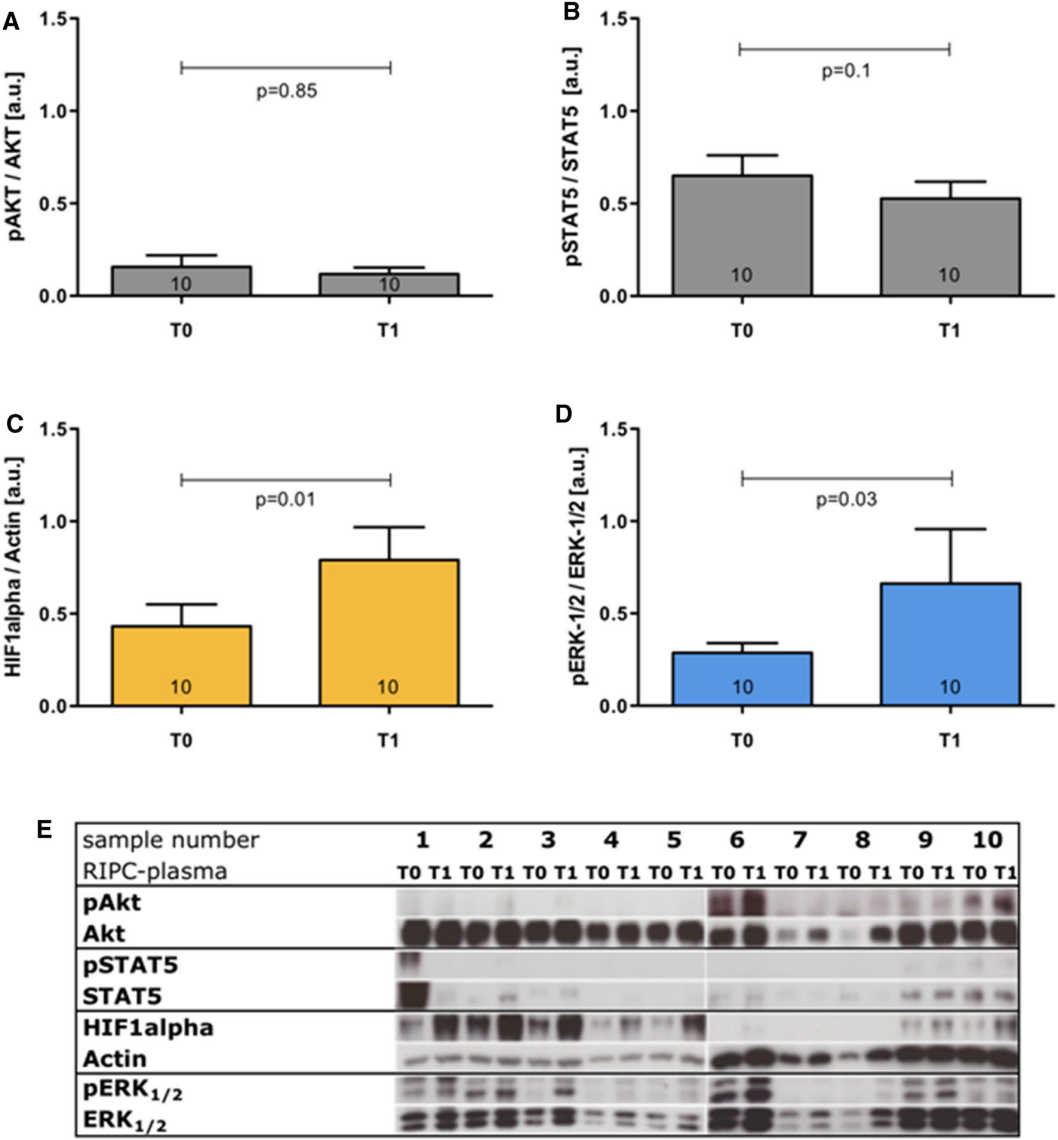
Effects of RIPC-plasma on protein expression and phosphorylation in HUVEC cells exposed to hypoxia. **a** Phosphorylation of AKT; **b** phosphorylation of STAT5; **c** expression of HIF1alpha; **d** phosphorylation of ERK-1/2. **e** Western blotting experiments performed with lysates of HUVEC cells that were treated with RIPC-plasma samples (#1–#10). *Numbers in the columns* show the numbers of different plasma samples used. *Columns* display the mean ± SEM

Using Western blotting, the expression of several proteins potentially involved in RIPC-mediated organ protection was investigated [21, 24, 39]. As only plasma *T*1 but not *T*2 was able to significantly reduce hypoxia–induced cell damage in HUVEC cells (Fig. 2), all Western blotting experiments were performed with HUVEC cells incubated with *T*1 and the respective control plasma (*T*0). Densitometric analyses revealed no statistically significant differences in the phosphorylation of AKT in HUVEC cells that were incubated with plasma *T*1 (*T*1: 0.12 ± 0.04 arbitrary units (a.u.) versus *T*0: 0.16 ± 0.1 a.u.; *p* = 0.85; Fig. 3a) or STAT5 (*T*1: 0.53 ± 0.1 a.u. versus *T*0: 0.65 ± 0.1 a.u.; *p* = 0.10; Fig. 3b). However, plasma *T*1 significantly augmented the amount of HIF1alpha (*T*1: 0.79 ± 0.2 a.u. versus *T*0: 0.43 ± 0.1 a.u.; *p* = 0.01; Fig. 3c) and increased the phosphorylation of ERK-1/2 (*T*1: 0.66 ± 0.3 a.u. versus *T*0: 0.29 ± 0.1 a.u.; *p* = 0.03; Fig. 3d) in HUVEC cells that were exposed to 24 h of hypoxia. Control studies revealed that the significant increase in HIF1alpha expression and the significantly increased phosphorylation of ERK-1/2 that were detected after the addition of protective plasma *T*1 was not evident when employing the non-protective plasma *T*2 (data not shown).

### Concentrations of VEGF are reduced in protective RIPC-plasma *T*1

**Fig. 4:**
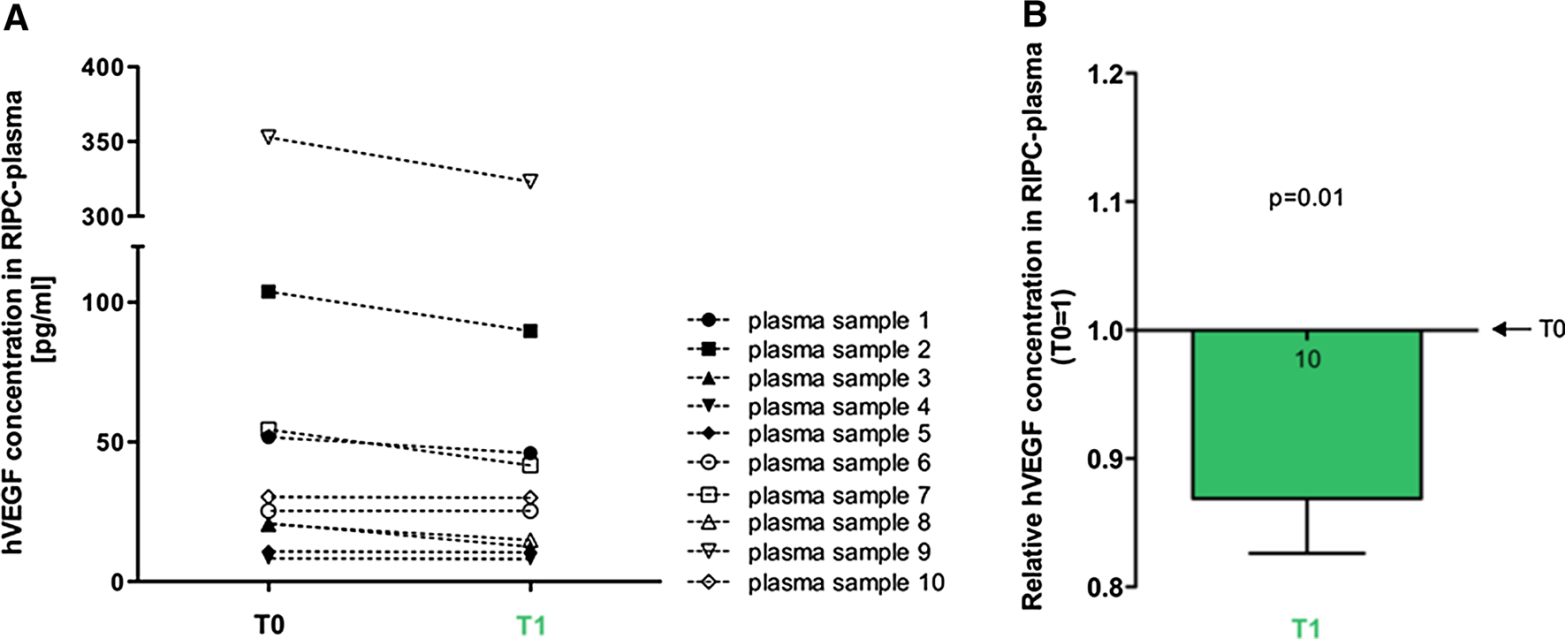
Quantification of hVEGF concentrations in human RIPC-plasma. **a** hVEGF concentrations in RIPC-plasma *T*0 and *T*1. **b** Relative hVEGF concentrations in *T*1-plasma. *Numbers in the columns* show the numbers of different plasma samples used. *Columns* display the mean ± SEM

As VEGF is discussed to be a humoral factor involved in RIPC-mediated organ protection [11, 13, 55], we evaluated the concentrations of human VEGF (hVEGF) in RIPC plasma using a sandwich ELISA system. Mean hVEGF concentrations in plasma *T*0 were 67.91 ± 32.9 pg/ml, while the range of hVEGF varied between plasma samples of different donors (minimum hVEGF: 8.26 pg/ml, maximum hVEGF: 352.80 pg/ml; Fig. 4a) Concentrations of hVEGF were significantly reduced in the protective plasma (*T*1) which was derived directly after RIPC (*T*1: 0.87 ± 0.04; *T*0 = 1; *p* = 0.01; Fig. 4b).

## Discussion

The major findings of the present study are the following: (1) human plasma retrieved directly after remote ischemic preconditioning (RIPC) is able to reduce hypoxia–induced damage of human endothelial cells cultured in vitro. (2) Expression of HIF1alpha but not phosphorylation of ERK-1/2, AKT or STAT5 seems to be involved in the protective effects of RIPC-plasma. (3) The protective RIPC-plasma contains decreased amounts of VEGF.

To date, the exact mechanisms of RIPC are not fully understood. However, three hypotheses to explain the phenomenon of remote ischemic organ protection have been established: (1) RIPC triggers the release of humoral factors into the bloodstream from where they reach the remote target organ; (2) neuronal pathways confer the RIPC-protection; and (3) a systemic anti-inflammatory and anti-apoptotic response is induced by the RIPC stimulus [24, 62]. Recently, several circulating mediators have been identified, e.g. stromal derived factor (SDF) 1alpha [6], exosomes [19], Apolipoprotein A1 [32], miR144 [45], IL-10 [8], or nitrite [60] that may be involved in RIPC-mediated cell and organ protection. Using an in vitro approach, we showed that serum from cardiac surgical RIPC patients as well as culture media from hypoxia–conditioned HUVEC cells are both able to reduce hypoxia–induced cell damage in intestinal cell cultures [36, 74]. These results underline the potential role of secreted factors for RIPC-mediated organ protection. Here we extended our recent studies and applied RIPC-plasma, which was retrieved from healthy male volunteers, to cultured endothelial cells.

In our study, plasma from RIPC volunteers (obtained before, directly after and 60 min after RIPC) was added to the HUVEC cell cultures 1 h before the hypoxic insult and cells were incubated with plasma-substituted medium for 24 h. It is known that ischemic preconditioning [42] represents a biphasic phenomenon with a first and a second window of protection [35] and similar mechanisms may also be effective in RIPC. The early phase of protection develops quickly within minutes from the initial ischemic conditioning event and lasts for 2–3 h. This is followed by a delayed phase that begins after 12–24 h and lasts up to 4 days. The mechanisms of the two phases of preconditioning are rather different. While the early phase is caused by rapid release or modification of pre-existing proteins, the delayed phase requires synthesis of new proteins [43, 44].

Our present findings showing cytoprotective effects of RIPC-plasma that was obtained directly after RIPC, but not of plasma derived 60 min after RIPC is somewhat in contrast to the above mentioned studies, clinical observations and also to our previous publication in intestinal cells (subjected to a hypoxic insult) [74]. However, in the frame of our previous study, RIPC sera were collected from mostly older cardiac surgical patients, while in the study presented here, 10 young and healthy donors were investigated. Several authors have shown that age, diet, hormonal status, comorbidities and other factors may influence and modify the protective potential of ischemic conditioning [1, 15, 17, 53]. Furthermore, the observation that only plasma that was derived directly after RIPC protected HUVEC cells from hypoxia–induced cell damage could be related to the half-life of the responsible factor(s). Potential mediators that might transfer the RIPC protection are adenosine [52, 61, 66], bradykinin [38, 61], opioids [67] as well as matrix MMPs [46, 73, 74] for review see [41], all of which have a limited half-life in circulation [54] and cell culture [18] and—especially in the case of MMPs—can be modified and/or degraded by other proteases [6, 73].

It should also be mentioned that while other authors employed serum [74], in the study presented we used plasma from RIPC treated volunteers. Compared to serum, plasma contains clotting factors such as fibrinogen but is deficient of mediators that are released from blood cells (mainly thrombocytes) upon coagulation. There is no evidence that these molecules interfere with RIPC-mediated processes and we therefore do not expect differences in the protective potential of serum in comparison to plasma.

Taken together, although we do not have a clear explanation why plasma obtained 60 min after RIPC was not effective in protecting HUVEC cells from hypoxia–induced cell damage in the study presented, individual characteristics (age, gender, hormonal status, diet, etc.) of the plasma donors and the use of an in vitro culture system (devoid of e.g. immune cells, humoral factors, blood circulation, etc.) may at least partially be responsible for this observation. Moreover, not all aspects of ischemia/reperfusion injury as they appear in vivo can be reflected using this in vitro system. However, the cell culture model enables us to reproducibly investigate isolated events of ischemia/hypoxia and the associated cellular as well as molecular mechanisms, which is probably the biggest advantage over animal and clinical studies.

Regarding the mechanisms that are induced by RIPC in the target cells, we found an increased amount of HIF1alpha protein in hypoxia stressed HUVEC cells that were treated with plasma derived directly after RIPC. HIF1alpha acts as an oxygen-regulated transcription factor controlling oxygen homeostasis [64] and activation of HIF1alpha leads to induction of target gene activation of e.g. erythropoetin, hexokinase 1 and 2, iNOS and VEGF [33]. Although little is known about the precise role of HIF1alpha in RIPC, several studies proposed an involvement of the protein in ischemic preconditioning [7, 25, 64]. However, there is also a study suggesting that upregulation of HIF1alpha in limb is not associated with myocardial protection of early RIPC and might only act locally [40]. Employing cardiac tissue of cardiosurgical patients that received RIPC or sham intervention, we recently showed that HIF1alpha expression was significantly increased in cardiac tissue of RIPC patients [3], pointing towards possible organ protective effects of enhanced HIF1alpha expression. The protective role of HIF1alpha is also supported by our preliminary experiments employing the HIF1alpha inhibitor LY294002 (10 µM) [72] which showed that the cytoprotective effects of plasma *T*1 are attenuated by inhibiting the translation of HIF1alpha.

Besides an enhanced expression of HIF1alpha, phosphorylation of ERK-1/2 was increased in hypoxia stressed HUVEC cells that were treated with plasma derived directly after RIPC. It is known that phosphorylation of ERK, being part of the pro-survival MAPK/ERK-pathway, results in inhibition of the pro-apoptotic Bad and is thus impeding the process of apoptotic cell death [20, 62]. In HUVEC cells, transient hypoxia can induce anti-apoptotic events and increase cell survival via ERK-dependent pathways [20] and in the porcine heart RIPC effects are also associated with augmented levels of phosphorylated ERK-1/2 [23]. Interestingly, our preliminary data employing the ERK kinase inhibitor PD98059 (10 µM) in combination with the protective plasma *T*1 did not confirm an involvement of ERK-1/2 in our in vitro setting, as the inhibition of ERK-1/2 phosphorylation did not result in increased cell damage measured by LDH activity. We propose that further work is necessary to elucidate the precise role of ERK-1/2 activation in RIPC-mediated cytoprotection, especially in endothelial cells.

Hausenloy et al. [23] not only reported pERK-1/2 to be involved in RIPC-mediated organ protection, but also pAKT. Similar to pERK-1/2, pAKT is involved in cellular survival pathways: the PI3 K/AKT-pathway, also known as “reperfusion injury salvage kinase (RISK) pathway”, phosphorylates and thereby inactivates the pro-apoptotic Bad leading to an inhibition of apoptosis [39, 62]. Interestingly, in the present study we did not observe an increased phosphorylation of AKT, which might indicate that different cell types (e.g. myocardial cells versus endothelial cells) respond differently to the RIPC stimulus.

In the literature, the involvement of STAT5 in RIPC is discussed controversially. While an increased phosphorylation of STAT5 was described in ventricular cells after RIPC in humans [31], other authors did not find alterations in STAT5 phosphorylation using cultured intestinal cells [36]. From recent studies it appears that STAT3 and STAT5 might have reverse functions in animals and humans: STAT5 but not STAT3 activation is associated with protection in humans [31], whereas STAT3 activation and possibly STAT5 inhibition are associated with protection in animals [30]. In a clinical trial with RIPC patients undergoing coronary artery bypass surgery, Heusch et al. [31] have shown that the phosphorylation of STAT5 increased from baseline before ischemic cardioplegic arrest to 10 min of reperfusion with RIPC, and that STAT5 phosphorylation during reperfusion was greater in patients with RIPC than in control patients. Once more, the target organ and/or target cell type might determine which signalling pathways are induced via RIPC and this might explain the lack of STAT5 phosphorylation in the context of our study.

Concerning possible factors transferring the RIPC signal to the target cells, VEGF could be a potential candidate [11, 13, 55]. Several studies suggested that VEGF reduces ischemic damage via ERK-1/2 dependent pathways [11, 13]. Surprisingly, in our study employing RIPC-plasma in combination with a cell culture system, VEGF specific ELISAs revealed significantly reduced levels of the protein in protective RIPC plasma. In the first place these data would suggest that VEGF is not involved in RIPC-mediated protection of endothelial cells. However, VEGF influences endothelial cell proliferation and migration and has been reported to stimulate the expression of metalloproteinases (MMPs) in HUVEC cells [4, 70]. We have recently shown that activities of MMP-2 and MMP-9 are reduced by RIPC in cardiac tissue of cardiosurgical patients [73]. These findings suggest that MMPs could be involved in RIPC and VEGF mediated mechanisms, however, further studies are needed to confirm a potential causal relationship between RIPC, VEGF and MMPs.

In conclusion, the results of the present study support the hypothesis that humoral factors confer RIPC-mediated cell and organ protection and we suggest endothelial cells as targets for RIPC-released mediators.
